# IMPLICATIONS FOR MORTALITY RISK: CONSEQUENCES OF SURVEY NONRESPONSE IN HOME-DWELLING OLDER ADULTS

**DOI:** 10.1093/geroni/igac059.544

**Published:** 2022-12-20

**Authors:** Marianne Razavi, William Dale, Colm O'Muircheartaigh, L Philip Schumm, Ashwin Kotwal, Linda Waite

**Affiliations:** City of Hope, Duarte, California, United States; City of Hope Comprehensive Cancer Center, Duarte, California, United States; University of Chicago, Chicago, Illinois, United States; University of Chicago, Chicago, Illinois, United States; University of California, San Francisco, San Francisco, California, United States; National Opinion Research Center (NORC at Chicago), Chicago, Illinois, United States

## Abstract

Nonrespondents generally suffer from worse health outcomes than respondents. Are they unwilling or unable to respond? Our aim was to address this issue. Data (N=3,130) from 2010-2015 waves of National Social Life Health and Aging Project (NSHAP, W2, W3) was used. Four groups of participants were considered based on their response status at W3: alive, incapacitated, deceased, and nonrespondents. Nonrespondents represented cases with no information at W3, beyond their disability and death information. General linear models were used to compare group means at baseline (W2) in terms of mortality risk (Lee index) or cognitive impairment (MOCA), adjusted for demographic variables. Like the deceased or incapacitated groups, the nonrespondent group displayed significantly worse outcomes (Least Squares Means) than the alive group: Lee index alive=5.82, deceased=9.66, incapacitated=8.29 and nonrespondents=7.80; MOCA alive=21.57, deceased=19.79, incapacitated=19.19 and nonrespondents=19.84. Being a nonrespondent likely indicates incapacity, not reluctance to responding. Earlier follow-up surveys could optimize response rates.

